# Experiences of a Motivational Interview Delivered by a Robot: Qualitative Study

**DOI:** 10.2196/jmir.7737

**Published:** 2018-05-03

**Authors:** Joana Galvão Gomes da Silva, David J Kavanagh, Tony Belpaeme, Lloyd Taylor, Konna Beeson, Jackie Andrade

**Affiliations:** ^1^ Cognition Institute School of Psychology University of Plymouth Plymouth United Kingdom; ^2^ Institute of Health and Biomedical Innovation Queensland University of Technology Brisbane Australia; ^3^ Centre for Robotics and Neural Systems University of Plymouth Plymouth United Kingdom

**Keywords:** robotics, counseling, motivational interviewing, motivation, exercise, qualitative research, computer-assisted therapy, person-centered therapy

## Abstract

**Background:**

Motivational interviewing is an effective intervention for supporting behavior change but traditionally depends on face-to-face dialogue with a human counselor. This study addressed a key challenge for the goal of developing social robotic motivational interviewers: creating an interview protocol, within the constraints of current artificial intelligence, which participants will find engaging and helpful.

**Objective:**

The aim of this study was to explore participants’ qualitative experiences of a motivational interview delivered by a social robot, including their evaluation of usability of the robot during the interaction and its impact on their motivation.

**Methods:**

NAO robots are humanoid, child-sized social robots. We programmed a NAO robot with Choregraphe software to deliver a scripted motivational interview focused on increasing physical activity. The interview was designed to be comprehensible even without an empathetic response from the robot. Robot breathing and face-tracking functions were used to give an impression of attentiveness. A total of 20 participants took part in the robot-delivered motivational interview and evaluated it after 1 week by responding to a series of written open-ended questions. Each participant was left alone to speak aloud with the robot, advancing through a series of questions by tapping the robot’s head sensor. Evaluations were content-analyzed utilizing Boyatzis’ steps: (1) sampling and design, (2) developing themes and codes, and (3) validating and applying the codes.

**Results:**

Themes focused on interaction with the robot, motivation, change in physical activity, and overall evaluation of the intervention. Participants found the instructions clear and the navigation easy to use. Most enjoyed the interaction but also found it was restricted by the lack of individualized response from the robot. Many positively appraised the nonjudgmental aspect of the interview and how it gave space to articulate their motivation for change. Some participants felt that the intervention increased their physical activity levels.

**Conclusions:**

Social robots can achieve a fundamental objective of motivational interviewing, encouraging participants to articulate their goals and dilemmas aloud. Because they are perceived as nonjudgmental, robots may have advantages over more humanoid avatars for delivering virtual support for behavioral change.

## Introduction

### Background

Lifestyle factors such as physical inactivity impose a considerable burden on society’s health care resources and individuals’ well-being [1]. Participants in qualitative studies focusing on weight management say they want motivational support to make lifestyle changes [2,3], but public health budgets constrain society’s ability to offer face-to-face counseling [4]. Social robots that can deliver effective motivational support could offer a way to increase access and encourage behavior change. This paper reports a study of participants’ experiences of a robot-delivered motivational interview to support their goal of becoming more physically active.

### Motivational Interviewing

Motivational interviewing (MI) [5] is one of the most effective psychological interventions for supporting behavior change [6,7], including for increasing physical activity (PA) [8,9]. The MI practitioner uses a person-centered counseling style to engage the client in discussion of their current problem and to elicit their own ideas for solutions. This collaborative stance is considered important, because people are likely to react to directive, advice-giving, (doctor-patient) counseling styles by trying to justify their current behavior [10,11]. The aim of MI is to encourage the client to voice their own arguments for change, as hearing oneself arguing for change increases belief that change is important and will happen [12]. Given the focus on personalized dialogue, MI delivered by a robot might seem a distant dream.

### Social Robots

Although people may have preconceptions about robots from science fiction films, few have had opportunities to interact with one. Two streams of development dominated early robotics: remote navigation for observing hard-to-reach environments and manipulation for replacing human manual work in industries. Recently, there has been a new focus on humanoid robots as personal assistants or carers in daily life [13,14].

These social robots have been used to provide educational support for children [15] and assistance to elderly individuals [16]. They have proven acceptable and effective for helping children with type 1 diabetes to learn about their condition and how to manage it [17] and are being trialed as therapeutic aids for children with autism spectrum disorders, with results showing therapeutic outcomes similar to those of one-to-one therapy [18,19]. Robots have also become personal trainers, instructing and motivating the completion of exercises such as spinning, rowing, and bodyweights [20] or engaging elderly users in physical exercises [21]. They have served as weight loss coaches, stimulating tracking of calorie consumption and exercise, and being twice as effective as a stand-alone computer or paper log [22]. However, naturalistic dialogue between robots and humans is currently limited by robots’ speech processing capabilities and the capacity of artificial intelligence to cope with unconstrained input [23]. The use of robots for therapy has therefore been limited to education and engagement rather than delivery of interventions where dialogue is critical.

### Using Technology to Deliver Adaptations of Motivational Interviewing

There have been attempts at mechanizing delivery of MI using text, audio, video, and animations, with some success [24]. For example, Jackson and colleagues used branching logic and a prerecorded Video Doctor to guide pregnant women through a motivational interview. Their trial showed improvements in diet and PA [25] and reductions in smoking [26], although no clear effects on smoking abstinence and weight were observed. There was also evidence that the Video Doctor led to more women discussing partner violence with their health care practitioner [27]. Interfaces have generally relied on participants entering text or selecting preprogrammed options, making the intervention less person-centered than is ideal and removing the benefits central to MI of hearing oneself argue for change.

### Social Robots as Motivational Interviewers

Social robots have the potential to engage participants in a motivational interview so that they hear themselves argue for a change. To our knowledge, only one other group has tested robots in this way. Kanaoka and Mutlu [28] used a NAO robot to deliver a motivational interview. They found no benefit of MI compared with a traditional advice condition. They attributed the lack of benefit of MI to a lack of fluency in the dialogue between the robot and the participant, with errors in speech recognition and incongruous nonverbal behaviors destroying the illusion of a meaningful two-way conversation. A complete motivational interview, with personally tailored questions and reflections upon the client’s answers, still poses substantial challenges to robot speech recognition and artificial intelligence.

This paper reports the development and assessment of a simpler solution, using a social robot to elicit change talk with a preprogrammed set of questions. In contrast to previous attempts to automate MI, apart from Kanaoka and Mutlu’s study, the focus of the interview was on encouraging participants to talk to the robot about their motivation for change, using open questions designed to draw attention to the discrepancy between the participant’s current behaviors and core values. Apodaca and Loganbaugh [29] found that change talk and experience of discrepancy are the main mechanisms of change in MI. A preprogrammed set of questions falls short of the person-centered counseling style that is at the heart of MI. However, if this approach succeeds in encouraging participants to talk freely about their concerns and their plans, we contend that it would present a substantial step forward in the use of technology to deliver motivational support.

The aim of the study was to explore participants’ experiences of talking to the robot in a dialogue based on MI but constrained by current technology. We specifically wanted to know how people felt about discussing their issues with the robot and whether they felt that the interview affected their motivation.

## Methods

### Motivational Interview Script

We created an intervention script using manuals developed for face-to-face motivational interviews in clinical trials [30,31] and Miller and Rollnick’s book *Motivational Interviewing: Preparing People for Change* [32] for guidance. Each question needed to make sense, regardless of how the participant answered the question before. To anticipate potential glitches in the flow of the dialogue, we iteratively role-played potential responses to the questions and adjusted the script where necessary.

We shaped participants’ expectations by advising them, at the start of the interview, that, “During this interview, sometimes I may ask you questions that you think you’ve already answered. If that happens, I suggest you use it as an opportunity to think about the issue a bit more.” The questions covered MI elements such as advantages and disadvantages of the status quo, optimism about change, intention to change, evocation of ideas about change, hypothetical change, setting goals, and arriving at a plan [32]. As in MI, the interview moved from a general discussion of the pros and cons of change to development of specific plans for change. The questions were designed to encourage participants to articulate their ideas about change and to consider the discrepancy between their current behaviors and core values. In a real motivational interview, the interviewer uses reflection as a tool for amplifying emotions associated with the pros and cons of change; repeating, paraphrasing, or elaborating salient statements made by the participant. Because this personalized reflection is not possible in a prescripted interview, we sought to amplify emotion using open questions to encourage the participant to think deeply about their incentives. For example, the robot asked, “What may happen in the future if you don’t change anything?” followed by “Does that worry or concern you?” The script did not refer to any specific goals or behaviors, so that it could be generalized to many situations, but participants knew that the study was about increasing PA. The full script is provided in Multimedia Appendix 1.

To help readers understand the strengths and weaknesses of the robot’s script, two of the authors trained in MI characterized it using Shingleton and Palfai’s [24] schema for rating technology-delivered adaptations of MI, which was published after we developed the robot interview. Shingleton and Palfai scored features of MI as present or absent. To give a more nuanced picture, we rated the degree to which each quality of MI was present, from 0 (absent) to 3 (fully present; see Table 1). We note that the standard tool for evaluating the quality of MI, the Motivational Interviewing Treatment Integrity manual [33], is not applicable here because it is used for rating the interviewer’s interaction with the client.

### Programming the NAO Robot

**Figure 1:**
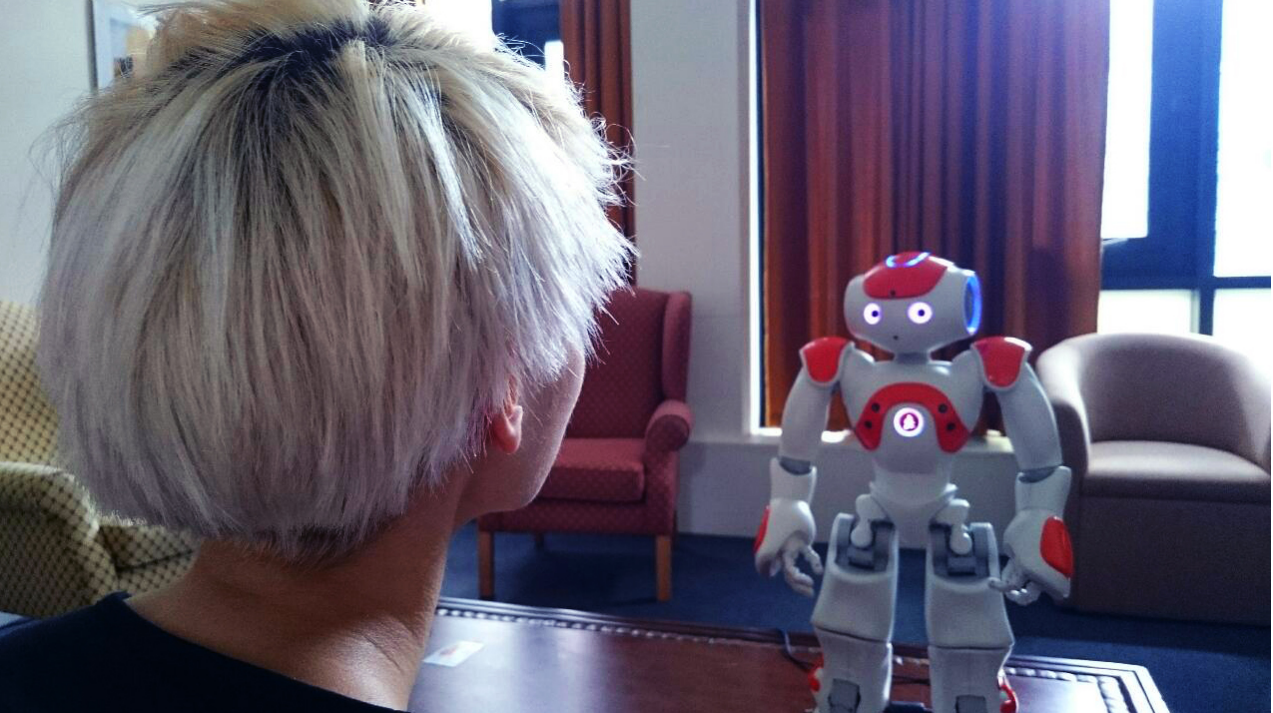
An illustration of the interaction between a participant and the NAO robot.

We used a NAO robot (Figure 1) to deliver this intervention. NAO is developed by SoftBank Robotics with speech and movement capabilities. It is brightly colored, 58 cm tall, with large eyes and humanoid appearance. NAO was chosen for this intervention because of its user-friendly software package Choregraphe and because it has been well received by participants in previous research [34]. The robot was programmed with Choregraphe software, which is used to create behaviors, monitor, and control the NAO robot. The instructions were sent wirelessly to NAO so that the experimenters were able to run the programmed script from a computer in a different room. The experimenters could monitor the progress of the interview visually via a live camera in NAO’s head.

Much of the programming effort was devoted to determining the intonation and speed of each sentence so that the questions were easy to hear. The robot’s voice pitch was kept at the default from the manufacturer. A simpler approach would be to record a human asking the questions and have the robot replay the recording. We felt this option was not viable because it would destroy the illusion that the robot was asking the questions itself. In addition to programming the robot’s speech, we incorporated some ready-programmed modules that come with the Choregraphe software package, to give the robot a life-like, animated appearance. These modules included breathing, in which the robot sways gently to emulate breathing and slight fidgeting, and eye color. The robot’s eye color changed from blue (question) to green (answer) to indicate whether the participant should listen or answer at each point in the interview.

**Table 1:** Characterization of the robot-delivered motivational interview using Shingleton and Palfai’s criteria for assessing technology-delivered adaptations of motivational interviewing (MI). Examples from the robot script are quoted in italics.

Rating	Components of motivational interviewing
0. Absent	Roll with resistance Structure adapted to readiness to change or interest or self-efficacy Express empathy
1. Present but superficial or inadequate	Promote autonomy, ask permission: *is it okay if we talk about this now?* Collaboration: *let’s focus on...* Other MI adherent behaviors: *how does that make you feel?* (amplifying emotion)
2. Present but not optimal	Develop discrepancy or explore ambivalence: *what may happen in the future if you don’t change anything?* Reflections or summary: summary was used— *I suggest you summarize what you are going to do...* —but reflection is not possible in a pre-scripted interview.
3. Fully present	Evocation: *why is that important to you now?* Promote self-efficacy: *what could you do, to make sure you follow your plan over the next week?* Strengthen commitment to change: *try summarizing the things that are likely to get better if you change your behavior* Open-ended questions: *what would be the first step?*

The face-tracking mode enabled the robot to follow the participant’s face, regardless of his or her movements during the interview, to give a sense that NAO was paying attention.

### Setting

For the interview with the robot, we utilized a laboratory (Figure 1), styled as a living room, to create a relaxed atmosphere. The participant was left alone with the robot in this room.

### Evaluative Questionnaire

A questionnaire was developed to explore participants’ experiences of their interview with the robot and their impressions of its impact on their motivation. We used an anonymous, computerized questionnaire rather than a semistructured interview because we wanted participants to feel as free as possible to give an honest account of their experiences and not feel socially pressured into praising the robot.

The questionnaire included 24 open questions. The questions were designed to address the primary aim of the study: to explore participants’ experiences of a motivational interview delivered by a social robot. To ensure that participants considered different aspects of the interview, questions asked about how they felt during the interview (eg, “How was your interaction with the robot?” and “How engaging did you find the interview with the robot?”), how easy they found the robot to use and understand (eg, “How was your understanding of each question? Was the content clear?” and “How did you find the robot’s interface? Was it easy or difficult to use?”), how they felt about listening to themselves discussing their goals aloud (because this is a core component of MI), and whether they perceived an impact of the interview on their motivation (“Did this interview with the robot affect your motivation? How?” and “Did you improve your physical activity after the robot interview? How?”). To encourage a balanced appraisal, two questions asked specifically what participants found the best, and worst, aspects of the intervention. The full questionnaire is provided in Multimedia Appendix 2.

### Participants

A total of 20 participants (17 female, 3 male) was recruited from the School of Psychology’s pool of research volunteers. Participants were required to be aged 18 years or above and received £8 per hour to participate. Eleven participants were aged 18 to 25 years, 4 participants 26 to 33 years, 1 participant 34 to 42 years, 2 participants 43 to 60 years, and 2 participants above 61 years.

The study advertisement asked for volunteers wishing to increase their PA. Participants were informed that they would take part in a PA intervention over two sessions, which included an interview with a robot. The study was approved by the University of Plymouth Faculty of Health and Human Sciences Research Ethics Committee, and informed consent was signed by participants, who were assured of anonymity and told they could withdraw at any point of the study as per British Psychological Society guidelines. They were told that the interview would take approximately 15 min and would not be recorded. Participants were advised not to take part in the study and to seek medical assessment if they had any concerns about their health or ability to exercise.

### Procedure

The robotic intervention was comprised of two phases: lab session I and lab session II. There was a 1-week interval between them to allow time for participants to reflect on any impact of the interview while minimizing forgetting and intervening variables.

In session I, participants answered the robot’s questions out loud in a simulated conversation with the robot, with the participants touching the robot’s head sensor to advance to the next question. As previously stated, NAO’s eye color changed from question to listening mode when it was the participant’s turn to speak. Participants were not alerted to this feature, as we intended it as a subtle turn-taking cue. Interviews were not recorded because participants in pilot work anticipated that they would feel uncomfortable talking to the robot as it was a novel experience and would prefer not to be recorded. We return to this issue in the Discussion.

One week later, in lab session II, participants returned to the lab and evaluated the intervention through a computerized evaluative questionnaire with open-ended questions and typed answers.

### Thematic Content Analysis

Participants’ answers to the evaluative questionnaire were content-analyzed utilizing a three step methodology recommended by Boyatzis [35]: (1) sampling and design, (2) developing themes and codes, and (3) validating and applying the codes. The first step of the content analysis was to define the set of units of analysis to be investigated further. The full set of responses to the qualitative questionnaire from each participant was delineated as each unit of analysis. There was a total of 20 units of analysis, one set per participant. The coding scheme originated from the text itself, with the main and subthemes being developed based on Boyatzis’ [35] steps: (1) generating a code, (2) reviewing and revising the code, and (3) determining the reliability of the code.

Immersive readings of the units of analysis led to the development of a series of potential codes. The text was further analyzed and generated a set of themes and subthemes based on common recurrent topics. Analysis continued until no new themes emerged.

In validating the code, it is important to check that the coding scheme can be applied consistently. Boyatzis recommends having two independent coders rate a subsample separately and computing the interrater reliability (IRR). We did this in two stages. First, two coders directly involved in the study (JGGdS and JA) rated two units of analysis independently. Differences in applying the code were discussed, and the coding scheme was adjusted accordingly. Then, two new raters, with no involvement in the study, applied the adjusted coding scheme to five further randomly selected units of analysis by deciding if each item in the code was mentioned or not. IRR was computed as the percentage of items agreed upon for each unit of analysis.

## Results

### Thematic Content Analysis

The coding scheme distinguished between experiences of interacting with the robot, participants’ own strategies for and barriers to motivation, PA in the week following the intervention, and overall evaluation of the intervention, which included suggestions for improvements. There was some similarity between the themes *interview evaluation* and *overall evaluation*. The interview evaluation theme incorporated answers to most of the questions and covered specific feelings experienced during the interview (for example, feeling relaxed, engaged, or self-conscious) and usability of the interface. The overall evaluation theme covered impressions of the intervention as a whole and suggestions for improvements, particularly but not solely covering responses to the questions about the best and worst aspects of the intervention. The theme on *motivation* covered ideas that participants used spontaneously, whereas the *PA* theme covered impressions of whether the interview affected motivation and activity in the week after the interview. The full coding scheme is shown in Multimedia Appendix 3.

Two experienced raters applied the coding scheme to two sample units of analysis. The IRRs for these units (P2 and P9) were 90% (52/58) and 97% (56/58), respectively. After slight adjustment of the coding scheme, two new raters, who were naïve to the purposes of the study, applied the adjusted coding scheme to five more units of analysis. Their mean IRR was 85.9% (249/290), ranging from 83% (48/58) to 91% (53/58). The coding scheme was assumed reliable.

### Participants’ Evaluation of the Intervention

#### Theme 1: Interview Evaluation

Participants’ evaluations of the interview clustered around four subthemes: how they felt about the interaction with the robot, their evaluation of the script, usability of the interface, and their experiences of hearing themselves speaking aloud to the robot.

##### Interaction or Connection With the Robot (1.1)

Most participants found the interaction smooth, felt relaxed or comfortable around the robot, and enjoyed the experience. Others found the experience interesting, unusual, or surreal. Most of the participants had an initial moment of tension followed by a period of relaxation after they became used to the robot. Although the novelty of being in proximity to a robot contributed to the initial awkwardness, it also added to the enjoyment of the experience, as illustrated below:

[My experience with the robot was] fine, if not a little awkward. The more time spent with the robot, the more relaxed I felt. [It was] easier to talk to than an actual person.

P2, age range: 18-25, female

It was a very novel experience as I had never been in such close proximity to a robot before and I certainly found it engaging.

P3, age range: above 61, female

I enjoyed interacting with the robot. It was like guided self-reflection. I was slightly nervous initially, but this soon passed and it became enjoyable.

P13, age range: 26-33, male

[It was] fun [talking to the robot]. It made me laugh to see its eyes change colour plus its squeaky voice was a giggle. After a while I forgot about the novelty of it all and just started to answer normally. Occasionally, though, it spouted out too much verbiage and I lost the plot. Over all, a good experience and one which will remain in my mind...It just felt like talking to a fun medical person—without the disinterested look of your average GP.

P15, age range: 43-60, female

For some participants, the lack of a personal response prevented them feeling connected with the robot:

I don’t think I interacted as I would have done [with] a human (I tend to look people in the eyes as I talk) as I didn’t feel a need to connect with it.

P2, age range: 18-25, female

However, this participant [P2] later identified advantages of the robot over a human interviewer:

Was easier to talk to than a human so suppose that made the conversation more engaging in that way as I felt able to open up more but really I didn’t feel as if the robot was interested in what I had to say, obviously.

Others also drew comparisons with talking to a human, and some preferred it because they felt they could talk without being judged:

Strange, felt like I was talking to a human. I have never experienced an interview with a robot before so it was an unusual experience.

P4, age range: 26-33, female

Possibly better than talking to a human as I wasn’t being judged eg with bored looks, bored body language, cutting words.

P15, age range: 43-60, female

...allowed you to be more honest as it’s not a human so no judgement.

P14, age range: 18-25, female

##### Script (1.2)

Most participants found the questions clear and easy to understand. Some had problems with some questions being too vague or ambiguous and having doubts about how to address them, although often they were not able to remember which questions had been problematic. Even though the robot warned at the beginning that it might sometimes ask questions that the participant had already answered, participants sometimes found it disconcerting or frustrating when this happened. This repetition could also be experienced as a positive feature. Some of the participants stated:

Quite engaging, particularly when a question came up that I felt I had already answered, as I would have to think about the topic a bit more in order to add something to my previous answer.

P13, age range: 26-33, male

At times I was confused as to how deeply the robot wanted me to answer the questions given, and so tended [I think] to delve too deeply as I was asked a few times to repeat what I had just said in another question. Did have one occasion where the automated voice sounded funny and wasn’t sure exactly what it had said!

P2, age range: 18-25, female

The content was clear, each question was clearly spoken...I found it frustrating that a question I may have already answered could be asked.

P5, age range: 18-25, male

The content was clear. Although I felt should have been more specific to the question. Questions sounded like they could relate to another subject generally so they were too generic and therefore less personal.

P6, age range: 26-33, female

##### Interface (1.3)

The instructions were clear and the navigation easy to use, and participants generally found it uncomplicated to touch the robot’s head sensor to advance to the next questions. However, some felt that this spoiled the illusion of a natural dialogue:

I felt a little concerned I might press something I should not and muddle up the process but it was fine.

P3, age range: above 61, female

Once I had stopped giggling at the eye colour change, everything was straightforward. Tapping on the head of the robot for the next question was simple.

P15, age range: 43-60, female

[The worst aspect of the interview was] not having the immediate response, having to push a button on his head made it feel fake.

P4, age range: 26-33, female

...It was extremely life like but having to tap it on the head to confirm you had completed your answer broke the rapport slightly. [The interface was] easy to use, it spoke clearly. It was good how his head followed your movement.

P6, age range: 26-33, female

##### Listening to Oneself (1.4)

Most of the participants found listening to themselves important. It helped them appreciate the importance of their goals and face the reality of their current behavior and plans for change. Some did not feel comfortable in speaking out loud and found the situation awkward. Some of the participants stated:

[Listening to myself was] very important. It’s easy to rationalise unhealthy behaviour in your head but the second you realise how stupid you sound rationalising or how reasonable your reasons are for wanting to do it, your attitude changes.

P2, age range: 18-25, female

[Listening to myself was] very important. Makes the thoughts hold more weight and actually think about them more than if they are simply passing thoughts.

P9, age range: 18-25, female

I regularly discuss behaviour with a team mate, so it is something that I consider is generally important. Usually when we discuss our behaviour, we critique errors and try to improve by correcting them. However, the robot also made me talk about times where my behaviour had been positive and this is something I think is very important.

P13, age range: 26-33, male

Actually, [listening to myself was] really rather important, as I could hear myself suggesting things, then getting a bit doubtful, then more confident as time went on. Hearing myself talking out loud made me feel as if I was chatting to myself and truly sorting out issues—without anyone else poking their nose in.

P15, age range: 43-60, female

I actually feel writing it down allows you to express yourself more honestly without the fear of sounding silly.

P6, age range: 26-33, female

#### Theme 2: Motivation

Participants’ spontaneous strategies (2.1) for supporting motivation included commitment or doing activities with friends or family; flexibility, routine, or planning; focusing on the goals; visualization techniques, mindfulness, or will power; and motivational books:

Using notifications on my phone to remind me when I have to do whatever it is I have to do [helps me staying motivated]. Also using diaries or planners to tick off when I’ve done it.

P2, age range: 18-25, female

[What helps me the most in staying motivated] is being mindful of the situation.

P3, age range: above 61, female

...visualising my goals and setting out steps I can achieve in the short term in order to achieve the long term goal.

P5, age range: 18-25, male

...Friends making supportive comments.

P15, age range: 43-60, female

Participants wrote about challenges (2.2) that make it hard to keep themselves motivated, including health problems, bad weather, winter, laziness or being tired, and social distractions:

[The hardest part in keeping myself motivated is] being distracted by something I shouldn’t be (like playing a video game for too long or watching another episode of something on Netflix).

P2, age range: 18-25, female

...tiredness, lack of time due to work. Winter when the days feel shorter.

P6, age range: 26-33, female

...erratic work shift patterns. Also, not seeing results within a certain timeframe can be demotivating.

P13, age range: 26-33, male

#### Theme 3: Engagement in Physical Activity After the Program

There was mixed success in terms of whether participants achieved their goal for the week after the robot interview. Some felt disappointed that they had not done so:

I didn’t improve my physical activity. It has been more or less the same as the past weeks.

P12, age range: 18-25, female

I did go for a run with a friend, as I said I would in the interview, however, this only happened once and so I feel it had not worked as well as maybe I had hoped.

P18, age range: 18-25, female

Others achieved their goals and occasionally expressed surprise in the way they communicated their success:

I actually carried out my plan...

P9, age range: 18-25, female

I stretched 3 out of 7 days and practised burlesque on 1, which is way more than I’d done regularly before.

P2, age range: 18-25, female

I completed at least 20 minutes of additional physical activity every day.

P4, age range: 26-33, female

#### Theme 4: Overall Evaluation

Participants’ positive appraisal of the intervention focused strongly on their perception that the robot was not judging them, whereas a human might have done. They liked being able to talk without being interrupted and appreciated how the interview gave them space to think about things and voice their goals. One participant described this as *a kind of liberation* [P12, age range: 18-25, female]. Some of the participants stated the following:

[The best aspect of this robotic interview was] being able to talk freely and for as long as I wanted about every aspect of physical activity that concerned me without being judged.

P2, age range: 18-25, female

...the time to talk without being interrupted.

P4, age range: 26-33, female

...he didn’t interrupt and was not judgemental...I felt more motivated because I talked through my goals without interruption or other people’s advice.

P10, age range: 34-42, female

The robot interview allowed me to reflect on my behaviour in a guided manner. It also encouraged me to focus on positive behaviour from the past and specific changes that I need to make for the future. I felt that this was the best aspect. When reflecting on my behaviour alone, there is a tendency to dwell on things done wrong and this does not always provide a solution. The robot demonstrated that I can reflect on my behaviour without focusing on negative aspects.

P13, age range: 26-33, male

The best part was the whole idea that I was able to interact with a robot. I think it feels nice to talk and not feel embarrassed by potential judgement.

P18, age range: 18-25, female

I felt I can talk freely without any judgement which was quite nice. Talking to “a human” is quite daunting as we naturally judge things and people especially people’s behaviour.

P19, age range: 18-25, female

The novelty of the robot was a positive feature for some. One participant explained how the fact that the interview was fun and memorable led her to share her goals with others. The robot may thus have contributed to that participant gaining “support from others,” which she cited as something that helps her stay motivated. Participants stated:

The use of a robot made it fun and less pressured which stayed in my mind longer...it played on my mind during the past week and I told others about the robot which made them ask about the goals set during the interview.

P1, age range: 18-25, female

It was engaging, different and fun...the fact I have thought about it over the past week has been motivational.

P3, age range: above 61, female

Participants offered insights into how the robotic interview could be improved. Common themes were the problem of not being able to replay a question that had not been understood, needing some time to get used to the robot, and wanting a more natural way of progressing to the next question:

I feel that the interview could be improved by having more off topic questions to begin with allowing the person to get used to the robot.

P5, age range: 18-25, male

[The robotic interview could be improved by] not having the robot to close, although that is essential to a certain extent, just felt awkward sitting so close—maybe it could be placed more to the side?

P2, age range: 18-25, female

Having to repeatedly touch the head for the next question was a little off-putting.

P9, age range: 18-25, female

Maybe it would be useful to have the robot repeating the question.

P12, age range: 18-25, female

[The worst aspect of this robotic interview was] having no feedback on my responses so I didn’t know if I was answering the question correctly.

P14, age range: 18-25, female

Perhaps a clearer voice. Sometimes, I felt I felt that I might have misunderstood a question due to not understanding the robot as well as I had wanted.

P18, age range: 18-25, female

## Discussion

### Principal Findings

We developed a technology-delivered adaptation of MI using a humanoid robot. When MI is translated into technology as a medium, this person-centered counseling technique inevitably loses its full capacity; however, we have developed a script with strong elements of MI, including evocation, promoting self-efficacy, strengthening commitment to change, and asking open questions. Key findings from participants’ evaluation of the intervention were that they found it motivating to hear themselves discussing their behavior with the robot; they enjoyed the interaction and found the robot easy to use but wanted longer to get used to it; and they liked the neutrality of the robot. The main drawback was that the robot could not tailor its questions according to the answers already given.

Previous research with technological delivery of MI has typically used text-based responses, for example, Gerbert’s work with the Video Doctor [25-27]. In a more ambitious project than ours, Kanaoka and Mutlu [28] used a NAO robot to deliver a motivational interview with personalized responses to the participant’s speech. In contrast to their predictions, participants were less motivated after the MI dialogue than after a monologue in which the robot gave advice. Kanaoka and Mutlu attributed this finding to inadequacy of the speech recognition software. They noted that the robot sometimes interrupted participants and that, when the robot “misheard” them, participants spoke to the microphone rather than to the robot, suggesting a breakdown in the fluency of the interaction. We tried to avoid these problems by using the robot to deliver a series of open questions and requiring the participant to press the robot’s head sensor when they had finished talking and were ready to advance to the next question. Participants evaluated this aspect of the interaction positively and negatively. They liked the space to talk freely about themselves, without interruption, and reported that the robot’s questions prompted them to think deeply and realistically about their goals and obstacles to achieving them. However, pressing the head sensor broke the flow of the conversation for some. The lack of personalization was frustrating, particularly when the robot asked a question that participants felt they had already answered. We had tried to preempt this problem by having the robot warn participants at the start that it might repeat a question. The interview deliberately asked several questions on one topic before moving to the next, to encourage the participant to think deeply about the issue and why it matters to them. This outcome would normally be achieved in MI through the interviewer reflecting the meaning of the participant’s answer back to them. Although participants typically disliked the repetition, one participant found that it helped him feel engaged in the dialogue by encouraging him to add more information to his previous answer.

An important aim of MI is to elicit change talk, where the individual articulates their desire or need to change. The extent to which a motivational interview elicits such talk is positively associated with outcomes [29]. In this study, participants found it motivating to hear themselves argue aloud for change, reporting that it helped them consolidate and take ownership of their plans. Many but not all participants felt that the interview had a positive effect on their behavior in the week that followed.

As most people do not have access to humanoid robots, the interaction with a NAO robot acting as a counselor was a unique experience. Due to the singularity of the situation, participants remembered the interaction and talked about it with other people, reiterating their commitment to change and making a social contract [36,37]. Further research is needed to test whether the effect of the interview fades away once the novelty wears off.

Although participants criticized the interaction for not being as fluent as a conversation with a human interviewer, some benefits of the robot interviewer featured very strongly in participants’ evaluations. They felt unhurried because the robot did not interrupt them, and many felt more comfortable discussing issues with the robot than with a human counselor because it would not judge them. A central tenet of MI is that interactions should be collaborative and not judgmental. These findings are an important reminder that, however skilled the interviewer, participants bring their own assumptions and anxieties to the interview, including a fear that the interviewer will judge them. In line with these findings, there is evidence that people will more willingly reveal sensitive information to computers than to humans [27,38,39]. In the drive to develop increasingly naturalistic computer-human interactions, developers must keep sight of the advantages of being perceived as a robot.

Participants spontaneously used a range of strategies to motivate themselves, including setting reminders, engaging peer support, having a routine, and visualizing their goals. As challenges to achieving their goals, they cited competing distractions, tiredness, and lack of time. There is scope for developing the robot interaction further to encourage successful behavior change strategies and reduce counterproductive behaviors. Previous work has shown that people can develop social relationships with robots [34]. Future research could explore the value of the robot for providing social support, which is known to facilitate behavior change, at challenging moments such as those mentioned by participants. This social support could include the ideas suggested by participants, such as reminding them of their plan, providing encouragement, or using imagery to strengthen motivation, for example, by guiding visualization of the goal and how good it will feel to succeed [40].

Participants wanted some time to get used to the robot before starting the interview. Providing a longer introduction before beginning the motivational interview could help address some of the drawbacks identified by participants, including discomfort at being close to the robot and having to touch it, and difficulty understanding its speech.

### Limitations and Directions for Future Research

Limitations of this approach include the impossibility of using all MI skills in a prescripted interview. Without more sophisticated speech recognition and branching logic, the robot is unable to reflect the participant’s meaning, affirm their choices and autonomy, or summarize what they have said (although we included suggestions, by the robot, that the participant summarize their plan). Even with perfect speech recognition, sophisticated MI skills, such as rolling with resistance and identifying change talk and sustain talk, present a considerable challenge. However, even without these skills, there is evidence that technological adaptations of MI can be beneficial [24]. There is potentially an issue of safety in terms of how a robot might respond to a participant who proposes a dangerous course of action. However, a skilled MI practitioner would elicit the participant’s appraisal of their plan, rather than directly advising against it, and this approach could be reproduced in the robot, as we did in this intervention through asking questions that probed an issue deeply before moving to the next. One solution to the problem of participants not knowing a safe solution to their dilemma could be for the robot to ask permission to offer advice or information. The NAO robot’s head, hands, and feet sensors also provide opportunities to follow different paths through the prescripted interview—for example, participants could choose information about diet by pressing a hand sensor or about exercise by pressing a foot sensor. Adding limited choice in this way may help to focus the interview on issues that matter most to participants and provide an experience that feels more personal.

The study has several limitations. It focused on participants’ experiences of the interaction and impressions of its impact on their behavior. Participants responded to an advertisement for volunteers who wanted to increase their PA, but we did not assess their motivation, baseline activity levels, or changes in behavior after the interview. Further research should test the robot interview with different populations, including those who wish to start being physically active and those who wish to increase their activity, and measure their pre- and postintervention motivation and behavior. Another limitation is that the people who volunteered for this study were members of a research panel and fairly used to strange experiences in psychology laboratories. They may have been more accepting of the robot, despite meeting it for the first time, than other members of the general population. Having established the acceptability of the intervention using qualitative methods, an important next step is to test its efficacy for changing behavior in a broader sample. Randomized controlled trials are needed to assess quantitative changes in motivation and PA associated with the robot intervention and compare them, in the first instance, against simple information and advice. To maximize the potential for observing benefits over meaningful timescales, we suggest that a series of interactions be designed to incorporate reminders and follow-up sessions so that the robot provides ongoing support for behavior change.

Much could be learned from observing the participant-robot interaction, but interviews were not recorded because participants who helped with the initial development thought they would feel uncomfortable being watched or filmed while talking to the robot. Having completed this study, we are less concerned that this would be an issue. A familiarization phase, with some general conversation between the participant and robot before starting the motivational interview, could help reduce the strangeness of the experience. In a related study evaluating a motivational interview delivered by a human video counselor, analysis of participants’ speech showed that the interview successfully elicited change talk (unpublished data, 2017 [41]). Combining analyses of change and sustain talk with quantitative data on behavior change could reveal whether a robot-led motivational interview affected motivation and behavior via the same mechanisms as human-led MI.

A robot-delivered motivational interview may lack elements of an interview with a human counselor, but our findings suggest it could have wide application. Because participants enjoyed the interaction and liked the novelty, a robot-delivered interview may help engage people to discuss sensitive issues and to get a feel for what counseling would be like, encouraging self-help or help-seeking earlier in the time course of a problem. A robot interview could be designed that encouraged people who are not yet contemplating change to consider its pros and cons. The novelty of interacting with a robot could encourage people to engage who might not feel ready to talk to a human counselor. Given that our adult participants were concerned about being judged by another adult, the robot could be particularly important for encouraging children and adolescents to discuss mental health issues, as they may be more susceptible to fears of being judged or misunderstood by an adult. As well as fostering engagement with health care, a robot interviewer could also provide motivational aftercare, ensuring that benefits from a human-led intervention are sustained when the intervention ends. The generic nature of the interview means it can easily be modified for a wide variety of target behaviors, potentially providing motivational support for the very large number of people who struggle with conditions such as addiction or obesity but do not meet the criteria for accessing professional support.

### Conclusions

We have shown, for the first time, that a motivational interview delivered by a social robot can elicit out-loud discussion from participants in an interaction that they perceive as enjoyable, interesting, and helpful. Participants especially found it useful to hear themselves talking about their behavior aloud, giving this new intervention a potential advantage over other technology-delivered adaptations of MI. Concern about being judged by a human interviewer came across strongly in praise for the nonjudgmental nature of the robot, suggesting that robots may be particularly helpful for eliciting talk about sensitive issues.

## Appendix

Multimedia Appendix 1 The motivational interviewing script delivered by the NAO robot.

Multimedia Appendix 2 The questionnaire used to explore participants’ experiences of the interview.

Multimedia Appendix 3 The coding scheme developed through the thematic content analysis.

## Abbreviations

IRR: interrater reliability
MI: motivational interviewing
PA: physical activity
