# Supplementary material for: Experiences of a Motivational Interview Delivered by a Robot: Qualitative Study
Source: J Med Internet Res. 2018 May 3;20(5):e116. doi: 10.2196/jmir.7737 (PMC5958282; doi:10.2196/jmir.7737)
Supplement: Multimedia Appendix 1 [file jmir_v20i5e116_app1.pdf]

Hi, my name is NAO.

Welcome to this interview with me!

After you answer each question, you will need to press the button to proceed to the next question.

Take your time to give a complete answer. The more you work on your answers, the more you can get out of this interview.

____

I understand that you are considering changing your behaviour, and you've already taken the first step by taking part in this interview.

During this interview, sometimes I may ask you questions that you think you've already answered. If that happens, I suggest you use it as an opportunity to think about the issue a bit more. Let's get started.

What have you been thinking about doing about your behaviour?

____

Why have you been thinking about doing that?

____

If you made this change, how will that affect other areas in your life?

____

What improvements might you notice in the first few days?

____

Let's put it this way--What may happen in the future if you don't change anything?

____

Does that worry or concern you? …Why?

____

Would anything else concern you if you keep doing things the way you are doing them?

____

Try summarising the things that are likely to get better if you change your behaviour.

____

When you think about that list of things, how does it make you feel?

____

OK—so, thinking about all the things that may get better if you made these changes, which is the most important one to you right now?

____

Why is that important to you now?

____

So, in thinking about all this, what goal might help you get these positive changes?

____

Let’s see if we can make this as specific as we can—what steps would you need to take, and when. What would be the first step?

____

Let’s focus on your confidence in getting started.

How confident are you that you can carry out this plan for the next week? Give it a rating from 0, not at all confident, to 100%, really confident.

____

Tell me about a time in your life when you had been doing better than you are now…

____

Can you remember other times that you did better?

____

Could you do something similar now?

____

Are there other times you’ve succeeded in changing your behaviour in the past—even for a short time?

____

Thinking about the things you did in the past to help you reach your goal, are there any strategies you could apply over the next week?

____

Are there other things you could do now, to help you get started?

____

Is there anyone who could help you over the next week?

____

Think about how you’ll carry out your plan over the next week. Chances are it will be a bit harder at some times than others. Is there a time in the next few days that may be a bit harder? Tell me about that.

____

What could you do, to make sure you follow your plan over the next week?

____

Sounds like a plan.

OK, let's redo your confidence rating.

How confident are you that you can carry out your plan for the next week? Remember, the rating scale goes from 0, not at all, to 100%, really confident.

____

Was your second rating a bit higher than the first one? Just thinking about a time you succeeded in the past, and how you did it, can often increase your confidence.

You don't have to be 100% confident to get started—you just need enough confidence to take the first step. Give it a go!

I suggest you summarise what you are going to do, why you want to do it, and what makes you confident you can at least do it for a week. What will you do? Why? And what makes you confident you can get started.

____

That's great. You may find it useful to write that down. If you need a bit of a boost to your motivation over the next few days, you could try reading that over to remind yourself about what you said.

All the best with your plans!

____
