# Supplementary material for: Experiences of a Motivational Interview Delivered by a Robot: Qualitative Study
Source: J Med Internet Res. 2018 May 3;20(5):e116. doi: 10.2196/jmir.7737 (PMC5958282; doi:10.2196/jmir.7737)
Supplement: Multimedia Appendix 2 [file jmir_v20i5e116_app2.pdf]

1. How was your experience during the robot interview?
2. How was your interaction with the robot?
3. How engaging did you find the interview with the robot?
4. How connected did you feel with the robot?
5. How was your understanding of each question? Was the content clear?
6. In case you had problems in understanding a question, do you remember which ones?
7. Did you answer all the questions? In case you didn’t, explain why and which question.
8. How were your feelings during the interview with the robot?
9. How was your mood in the day you had the interview with the robot?
10. How did you feel about hearing yourself talk about your goals out loud?
11. How important do you think it was listening to yourself out loud discussing your behaviour?
12. Were the instructions regarding the robot interview clear?
13. How did you find the robot's interface? Was it easy or difficult to use?
14. Did you get frustrated at any point during the interview? Why?
15. Would you use a robot like this in future to help you keep motivated?
16. What’s the best aspect of this robotic interview for you?
17. What’s the worst aspect of this robotic interview for you?
18. How do you think the robotic interview could be improved?
19. Have you tried other motivation techniques before?
20. What helps you the most in staying motivated?
21. What is the hardest part in keeping yourself motivated?
22. Did this interview with the robot affect your motivation? How?
23. Have you adopted any strategy to motivate yourself in the past week?
24. Did you improve your physical activity after the robot interview? How?
