# Supplementary material for: Experiences of a Motivational Interview Delivered by a Robot: Qualitative Study
Source: J Med Internet Res. 2018 May 3;20(5):e116. doi: 10.2196/jmir.7737 (PMC5958282; doi:10.2196/jmir.7737)
Supplement: Multimedia Appendix 3 [file jmir_v20i5e116_app3.pdf]

Hierarchy	Themes and sub-themes
**1**	**Interview Evaluation**
**1.1**	**Interaction/Connection with the robot**
1.1.1	Positive
1.1.1.1	Smooth connection, enjoyable experience, feeling relaxed, comfortable or cheerful
1.1.1.2	Engaging, interesting, fascinating or helpful
1.1.1.3	Better than talking to a real person or writing
1.1.1.4	Other
1.1.2	Neutral
1.1.2.1	Surreal, unusual or novel experience
1.1.2.2	Other
1.1.3	Negative
1.1.3.1	Not engaging, feeling unconnected, strange or awkward
1.1.3.2	Better to write or speak to a real person
1.1.3.3	Concerns about how to operate the robot
1.1.3.4	Self-conscious of being filmed or feeling shy
1.1.3.5	Difficulty in understanding the robot sometimes
1.1.3.6	Other
**1.2**	**Script**
1.2.1	Positive
1.2.1.1	Clear
1.2.1.2	Other
1.2.2	Negative
1.2.2.1	Repetitive
1.2.2.2	Impersonal, generic, or ambiguous
1.2.2.3	Other
**1.3**	**Interface**
1.3.1	Instructions
1.3.1.1	Clear
1.3.1.2	Other
1.3.2	Navigation
1.3.2.1	Easy to use
1.3.2.2	Other
**1.4**	**Listening to oneself**
1.4.1	Positive
1.4.2	Neutral
1.4.3	Negative
**2**	**Motivation**
**2.1**	**Secondary Strategies**
2.1.1	Commitment or doing activities with friends or family
2.1.2	Flexibility or routine or planning
2.1.3	Focus on the activity or goals (reports, reminds, results)
2.1.4	Visualization, mindfulness, or will power
2.1.5	Good weather
2.1.6	Motivational books
2.1.7	Reminders or planners
2.1.8	Other
**2.2**	**Challenges**
2.2.1	Health problems
2.2.2	Bad weather or shorter days on winter
2.2.3	Laziness or being tired
2.2.4	Social distractions, other things to do, setting for less, lack of time or money or not seeing results
2.2.5	Consistency

2.2.6	Other
**3**	**Engagement in physical activity after the program**
3.1	Didn’t increase exercise
3.2	Planned and executed activities, increased exercise, or intensity
3.3	Increased activity, but didn’t meet goal
3.4	Other
**4**	**Overall evaluation**
**4.1**	**Suggestions**
4.1.1	Not having the robot too close
4.1.2	Not having to press the button
4.1.3	Initial questions to get used with the robot
4.1.4	The robot could speak slower, repeat question or encourage to speak more
4.1.5	Lifelike or clearer voice
4.1.6	More specific questions or individualised response
4.1.7	Material could be seen by a specialist
4.1.8	Other
**4.2**	**Positive appraisal**
4.2.1	Space to think about things or voicing goals
4.2.2	Helpful making plans or increased motivation
4.2.3	Robot was fun, cute or entertaining
4.2.4	Non-judgemental aspect or not being interrupted
4.2.5	Innovative concept
4.2.6	Nice setting
4.2.7	Positive having face-tracking mode
4.2.8	Other
